# Analysis of Genome Sequences of Coagulase-Negative Staphylococci Isolates from South Africa and Nigeria Highlighted Environmentally Driven Heterogeneity

**DOI:** 10.7150/jgen.53019

**Published:** 2021-05-13

**Authors:** Tawanda Elias Maguvu, Adegboyega Oyedele Oladipo, Cornelius Carlos Bezuidenhout

**Affiliations:** Unit for Environmental Sciences and Management: Microbiology, North-West University, Potchefstroom, South Africa. Private Bag X6001, Potchefstroom, 2520, South Africa

**Keywords:** Antibiotic resistance, Coagulase-negative staphylococci (CoNS), environmentally driven heterogeneity, Pan-genome analysis.

## Abstract

Here, we report high-quality annotated draft genomes of eight coagulase-negative staphylococci (CoNS) isolates obtained from South Africa and Nigeria. We explored the prevalence of antibiotic resistance and virulence genes, their association with mobile genetic elements. The pan-genomic analysis highlighted the environmentally driven heterogeneity of the isolates. Isolates from Nigeria had at least one gene for cadmium resistance/tolerance, these genes were not detected in isolates from South Africa. In contrast, isolates from South Africa had a *tetM* gene, which was not detected among the isolates from Nigeria. The observed genomic heterogeneity correlates with anthropogenic activities in the area where the isolates were collected. Moreover, the isolates used in this study possess an open pan-genome, which could easily explain the environmentally driven heterogeneity.

## Introduction

Coagulase-negative staphylococci (CoNS) species have traditionally been considered commensals, however, many CoNS species are now being recognised as potential opportunistic human pathogens. Several studies have reported CoNS species being the most frequently isolated organism from bloodstream infections in intensive care units 1, 2. Though less virulent than the *Staphylococcus aureus* complex, CoNS are an important reservoir of antibiotic resistance genes and virulence factors that are transferred to closely related species such as *S. aureus* 3. This results in the evolution and emergence of successful clones of methicillin-resistant *S. aureus* (MRSA) 3. MRSA are multidrug resistant and are resistant to the last resort β-lactam, armamentarium 4. As a result, costs, length of hospital stay, as well as mortality resulting from infections associated with MRSA, are unsurprisingly high 5, 6. Besides aiding MRSA through horizontal gene transfer, several outbreaks of multidrug antibiotic-resistant CoNS have been reported 7, 8, 9. Methicillin-resistant CoNS (MR-CoNS) have a high genetic diversity particularly in genes conferring methicillin resistance 10, 11. Thus the knowledge about genetic characteristics and variability of the species (CoNS) is really important to assess.

Here, we report high-quality draft genomes of eight CoNS isolates from two distinct geographic areas (North-West province, South Africa, and Ile Ife, Osun State, Nigeria), with distinct selective pressures on the microbes. Elevated levels of tetracycline and other antibiotics have been detected from wastewater treatment plants in the North-West province, South Africa, suggesting the overuse of these antimicrobials 12. A number of studies reported extreme soil and surface water contamination with heavy metals (including cadmium) in and around Ile-Ife, Osun State, Nigeria 13, 14, 15, 16. Thus, we hypothesised that these anthropogenic activities might drive genome modifications which could be reflected in the accessory genomes. We used bioinformatics platforms to analyse the genomic features of the genomes, as well as exploring the antibiotic resistance and virulent genes associated with the isolates. We highlighted environmentally driven heterogeneity among the genomes, augmenting how anthropogenic activities are impacting the microbes in our surroundings.

## Materials and methods

### Sample collection

Coagulase-negative staphylococci (CoNS) isolates were sampled from wastewater treatment plants in North-West province, South Africa as well as in Ile-Ife, Osun State, Nigeria. Supplementary Table 1 provides information on the samples including the created IDs and the country of origin from which the samples were collected. Isolation and preliminary identification of the isolates were done as previously described 17. Briefly, after filtration of the wastewater, filters were plated on blood agar and mannitol/salt agar (MSA), and incubated for 24 - 48 h at 37 °C. Growth and fermentation of mannitol on MSA, as well as haemolysis on blood agar were examined and noted in all the samples. Presumptive staphylococci isolates were identified based on morphology and biochemical tests (Gram-positive cocci formed in clumps), catalase, coagulase, and DNase tests 18. CoNS isolates were identified by API STAPH (bioMérieux) following the manufacturer's protocols. In addition, we included clinical samples provided by the Microbiology Laboratory of Ile-Ife Seventh-Day Adventist hospital, an exact geographical location as the environmental samples from Nigeria. We expected the clinical samples to be distinct from environmental samples, however, we hypothesised that anthropogenic activities in the area (cadmium pollution) will likely be reflected in both environmental and clinical isolates.

### Genomic DNA extraction and sequencing library preparation

Bacterial DNA was extracted from overnight Muller-Hinton broth cultures using the Nucleospin® Tissue extraction kit (Macherey-Nagel, Düren, Germany) following the manufacturer's protocol. Agarose gel electrophoresis and NanoDrop spectrophotometry (ND-100, NanoDrop Technologies Inc, Wilmington, DE, USA) were used to determine the integrity and the purity of the resultant DNA, respectively. Paired-end libraries were prepared from 1 ng bacterial DNA using Nextera XT DNA Sample Preparation Kit and Nextera XT Index kit (Illumina Inc., San Diego, California, USA) following the manufacturers protocol. Sequencing of all the isolates shown in Supplementary Table 1, was performed using a MiSeq 2000 Illumina platform (250bp paired-end reads).

### Genome assembly, functional annotation, and downstream analysis

Raw sequence reads were quality filtered and trimmed using FastQC (http://www.bioinformatics.babraham.ac.uk/projects/fastqc/) and Trimmomatic 19, with the average quality set at Q15. *De novo* assembling of the quality reads was performed using SPAdes version 3.13.0. 20, 21 with minimum contig length set to > 10 000 bp. CheckM 22 was used to assess the quality of the SPAdes assembled genomes. SPAdes assembled genomes were annotated by both Prokka 23 and RAST 24 with default settings. The annotated genomes were inserted into a species tree using FastTree version 2.1.10 25. To determine the identity of the species, FastANI version 0.1.2 26 was used to estimate the Average Nucleotide Identity (ANI) of the isolates using nearest genomes from the phylogenetic tree as reference genomes. Kbase App 20, was used to compute pan-genomes and generating pan-genome circle plots.

### Identification of mobile genetic elements, virulence factors, and antibiotic resistance genes

Integrated prophage regions were identified and annotated by using the online PHAge Search Tool Enhanced Release (PHASTER) 27 with default parameters. PHASTER provides the prophage region length, its GC content as well as its position on the contigs. In addition, it estimates the intactness (complete/incomplete) and the most common related phages. Plasmid identification was done using PlasmidFinder 2.1 28 with default parameters. Virulence factors were identified using the Virulence Factors of Pathogenic Bacteria Database (VFDB) 29 using *S. aureus* subsp aureus as a genome for comparison. The Comprehensive Antibiotic Resistance Database (CARD) 30 was used to find the antibiotic resistance genes from the isolates using the perfect and strict hits only criteria. CRISPRCasFinder 31 was used to detect clustered regularly interspaced short palindromic repeats (CRISPR) from the genome sequences.

## Results

### Genomic features of the Staphylococcus isolates

Assembling of the genomes with SPAdes resulted in high quality draft genomes shown in Table 1. CheckM quality assessment of the assembled genomes showed that the isolates had an average of 97.9 ± 2.4 % completeness, having average contamination of 0.48 ± 0.43 % (Supplementary Table 2). Three of the isolates (T28, T27, and T28b) had no detectable contamination. On average, 30.25 ± 17 contigs per genome were detected and this corresponds to an average N50 of 289, 514.4 ± 220,373.6. The isolate L4 (*S. haemolyticus*) had the smallest genome size (2,135,828) corresponding to an N50 of 41,586. This genome had the largest number of contigs (60). In contrast, T28b (*S. cohnii*) had the largest genome size (2,690,294) corresponding to an N50 of 566,475, having a joint least number of contigs (12) with the isolate T28 (*S. cohnii*) (Table 1.) The G + C content was homogeneous in all isolates averaging 32.41 ± 0.27 %. The Prokka pipeline was used for functional annotation predicting on average 2,489.8 ± 163.7 genes. Isolate L4 had the least number of predicted genes 2,162 whereas isolate D13b had the highest number of predicted genes 2,658. On average, the number of protein-coding genes was 2,444.9 ± 173.7.

### Phylogenetic relationship with other Staphylococcus isolates

**Figure 1:**
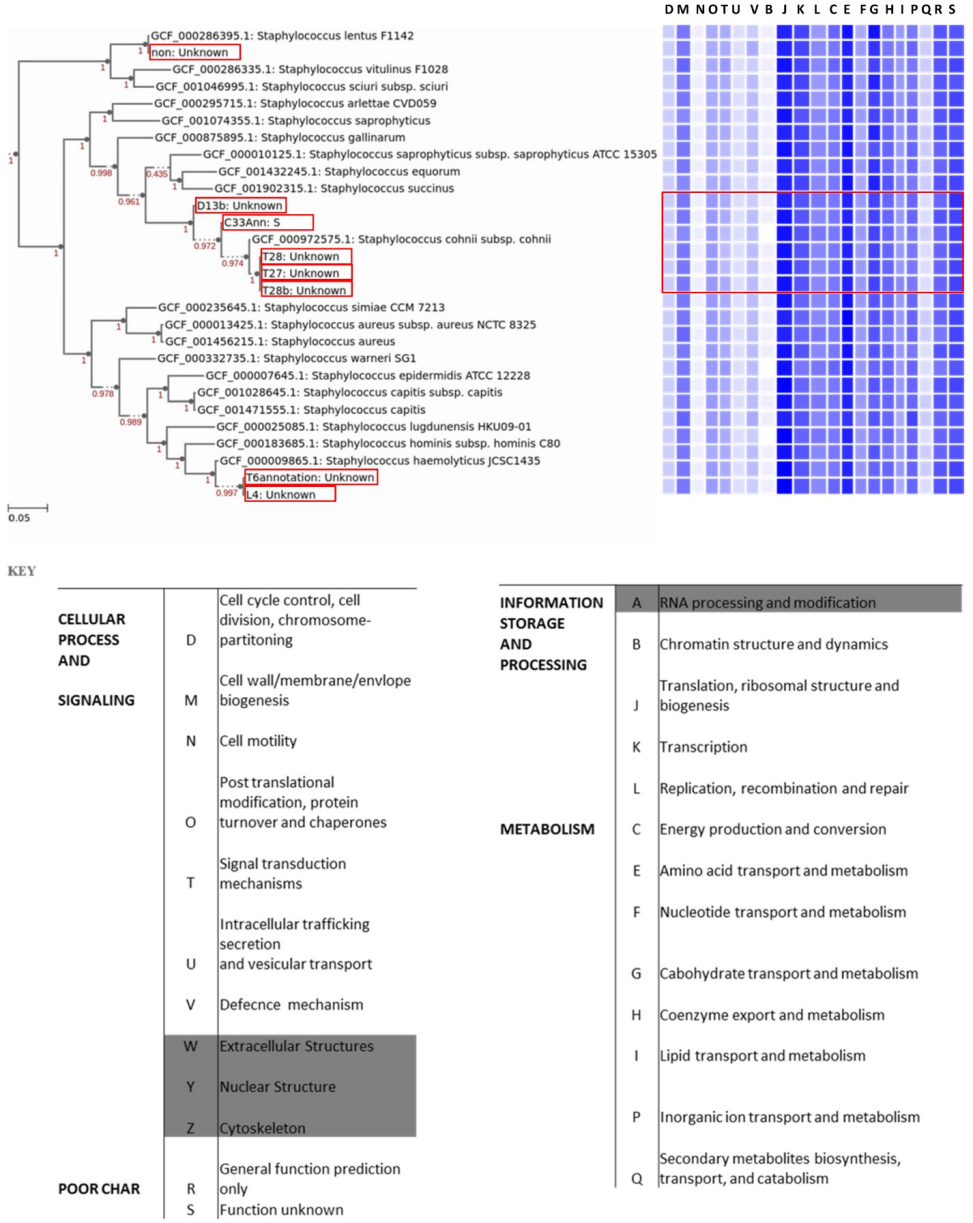
** Distribution of the Cluster of Orthologous Groups (COGs) function among the isolates and closely related species**. The COGs were generated from the tree's core genes, annotation of the genomes was performed using RAST. Isolates from this study are highlighted in red; from top to bottom the isolates are T20, D13b, C33, T28, T27, T28b, T6, and L4, respectively. Letters D-S on top of the heat map which corresponds to letters D-S on the key represents COG categories shown on the key.

The annotated genomes were used to construct a phylogenetic tree with closely related, publicly available genomes. Core genomes of the species shown on the tree (Fig. 1) were used to determine the distribution of a selected subset of Cluster of Orthologous Groups (COGs) functional domains shown in Fig 1. The core gene category repartition was highly similar among all the isolates and the closely related species (Fig. 1). Our sequenced genomes were identified as *S. lentus* (T20), *S. cohnii* (D13b, C33, T27, T28, and T28b), and *S. haemolyticus* (T6 and L4) (Fig. 1). To validate the assigned species of the isolates, FastANI was used to compute the Average Nucleotide Identity (ANI) of orthologous gene pairs between the query and reference genomes. Same species are considered to have an ANI of > = 95% 27. *Staphylococcus lentus* F1142 (GCF_000286395.1) when used as a reference genome had an estimated ANI of 99.1546 % with the query T20 (Table 2). They also had an approximately similar genome size of 2.5 Mb (Supplementary Fig. 1). Reference genome *S. cohnii* subsp. cohnii (GCF_000972575.1_assembly) had an estimated ANI of at least 98.7 % with any of (T28, T28b, T27, C33, and D13b) as a query (Table 2). Moreover, they had approximately the same genome size of 2.5 Mb (Supplementary Fig. 1). Reference genome *S. haemolyticus* JCSC1435 (GCF_000009865.1_assembly) had an estimated ANI of at least 98.8% with any of (T6 and L4) as the query. However, T6 and L4 had an estimated smaller genome size of 2 Mb in comparison with the reference genome size of 2.5 Mb (Supplementary Fig. 1).

### Pan-Genome Analysis

**Figure 2:**
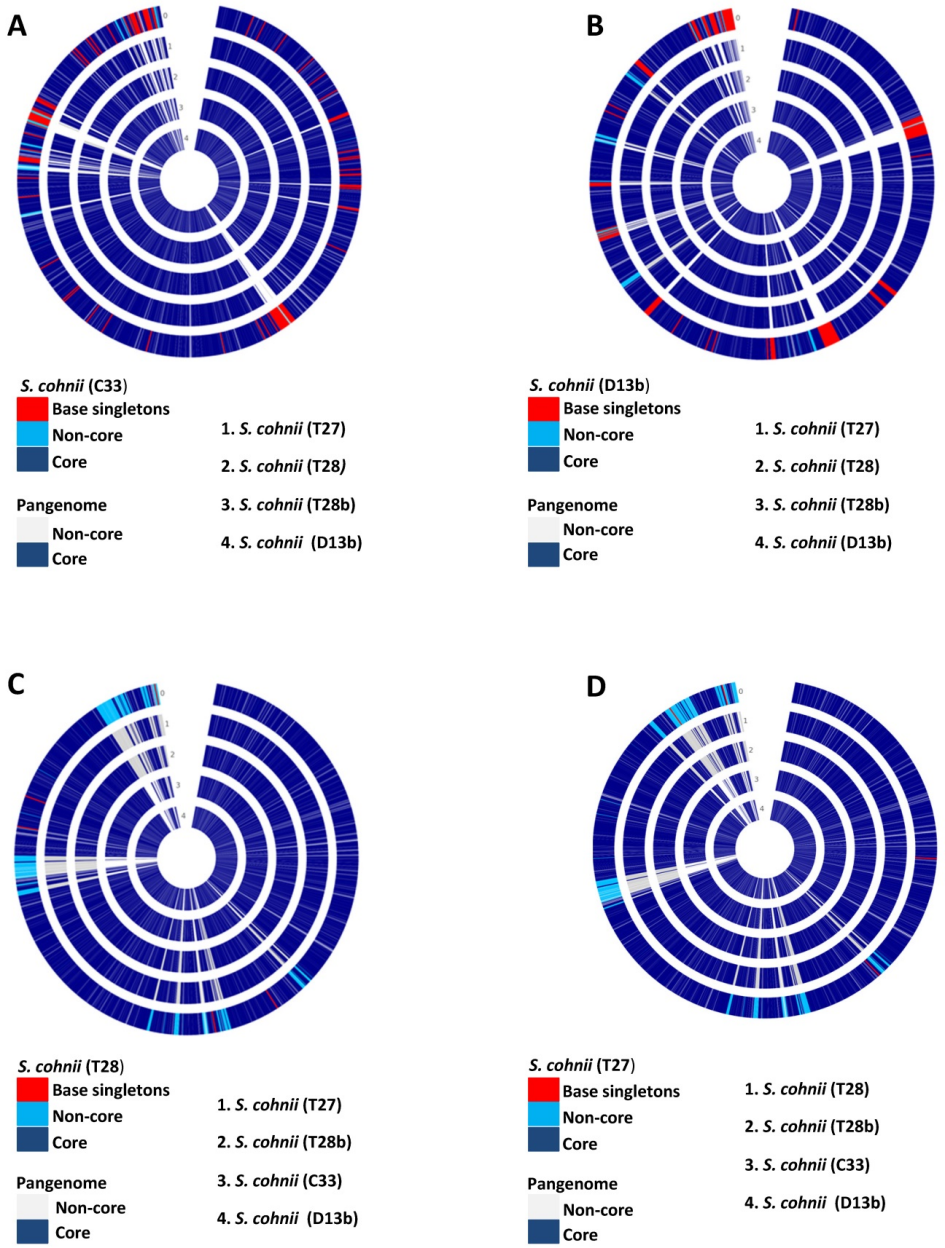
** Pan-genome circle plots of the *S. cohnii* isolates used in this study**. (A) Isolate C33 is the base genome being compared with the genomes of isolates (T27, T28, T28b, and D13b). (B) Isolate D13b is the base genome being compared with genomes of isolates (C33, T27, T28, and T28b). (C) Isolate T28 is the base genome being compared with genomes of isolates (D13b, C33, T27, and T28b). (D) Isolate T27 is the base genome being compared with genomes of isolates (T28, T28b, C33, and D13b). 0 depicts the base genome on all the plots.

The pan-genome analysis allows for better identification of diversity and composition of gene repertoire within species. The pan-genome is divided into three: (1) the core genome; which is a set of all genes common to all strains of the study, (2) non-core/accessory genome; which is a set of genes present in more than one, but not in all of the strains used in a study, and (3) singletons; which are genes unique to individual strains used in the study. A total of five isolates (D13b, C33, T27, T28, and T28b) were identified as *S. cohnii*, thus to have a higher resolution of the variability among these genomes, we applied the pan-genome analysis approach. Isolates (D13b and C33) had 240 and 180 singletons, respectively. In contrast, isolates (T27, T28 and T28b) had 4, 5, and 23 singletons respectively. Fig. 2 depicts the output from pan-genome analysis of the isolates identified as *S. cohnii*. The singletons for the isolate D13b were composed of bacteriophages, phage related genes, restriction-modification (RM) proteins and genes (Type I, II, III restriction-modification system), metal tolerance and resistance as well as stress response (Sensor protein DegS, respiratory nitrate reductase gamma chain, nitrate reductase, Multicopper oxidase, cadmium efflux system accessory protein, cadmium resistance protein, cadmium-transporting ATPase among others), several virulence determinants (Integrase, superantigen-encoding pathogenicity islands SaPI, Putative EsaC protein analog Listeria type 3), and a substantial amount of the singletons were predicted genes of unknown functions as well as hypothetical proteins. The functional distribution of the core genome is shown in Fig. 1, and it consisted of genes involved in metabolism, cellular process and signalling, information storage and signalling as well as poorly characterised functions.

In isolate C33, the singletons covered almost similar categories to D13b, which are resistance and tolerance to stress, virulent and pathogenicity determinants with a substantial amount of hypothetical proteins, and genes of unknown functions. Some of the genes worth mentioning are Multidrug and toxin extrusion (MATE) family efflux pump YdhE/NorM, homolog, Cassette chromosome recombinase B, Antiadhesin Pls, binding to squamous nasal epithelial cells, Arsenical resistance operon repressor, PlcR-regulated protein PRP2, Na+/H+ antiporter, SceD-like transglycosylase, biomarker for vancomycin-intermediate strains. The isolate T28b had 23 singletons with Bacitracin transport ATP-binding protein bcrA, Universal stress protein family and, Fosfomycin resistance protein FosB among the singletons. In isolate T27 and T28 singletons were mainly of unknown function. In all isolates from South Africa, *tetM* gene was detected (part of the accessory genome), but it was not detected in any of the isolates from Nigeria (see Table 3). In addition, *S. cohnni* isolates from Nigeria had at least one gene for cadmium resistance in their accessory genome or singletons, these genes were not detected in isolates from South Africa. Isolates L6 and T4 were identified as *S. haemolyticus*, though they were also from distinct geographic origins, we did not do the pan-genome analysis because their genomes were estimated to be incomplete.

### Mobile Genetic elements

Two intact prophage regions were identified by PHASTER analysis for two (L4 and C33) of the eight isolates. L4 and C33 prophage regions had a total length of 40.1kb and 58.5kb, encoding 59 and 75 proteins, respectively. For L4 the most common phage name was PHAGE_Staphy_StB12_NC_020490, whereas for C33 it was PHAGE_Staphy_phiRS7_NC_022914. Of the proteins encoded by L4 prophage region, peptidoglycan hydrolase, Erf-like recombinase, putative cro/cl-like repressor, LexA repressor, pyrophosphatase, pathogenicity island protein, and mobile-element-associated regulatory protein were of interest. In contrast, C33 encoded holin, dihydrofolate reductase type 1, protein phosphatase, and serine/threonine kinase.

PlasmidFinder detected a total of nine plasmids distributed among five of the eight isolates (Table 1). D13b plasmid was identical to *S. epidermidis* plasmid SAP016A (GQ900381), while that of T6 was identical to *S. epidermidis plasmid* SAP105A (GQ900452). Isolate T27 and T28 had two similar plasmids which were identical to *S. saprophyticus*. subsp saprophyticus ATCC 15305 plasmid pSSP1 (AP008935) and Staphylococcus sp. 693-2 plasmid pLEW6932 (NC_009130.1). The isolate T28b had three plasmids identical to *Staphylococcus aureus* subsp. aureus ST398 pS0385-3 plasmid, isolate SO385 (AM990995.1), *Staphylococcus saprophyticus* pSES22 plasmid, isolate 44 (AM159501.1) and *Staphylococcus saprophyticus* subsp. saprophyticus ATCC 15305 plasmid pSSP1 (AP008935.1). We were mainly interested in virulence and antibiotic resistance genes carried on the plasmids or mobile elements since they can be easily transferred to other microbes in the environment. Only T28b plasmid carried a gene of interest (*FosB*), which was on the plasmid identical to *Staphylococcus saprophyticus* subsp. saprophyticus ATCC 15305 plasmid pSSP1 (AP008935.1). Though no plasmid was detected for T20, the genome had an insertion sequence (IS256-like element ISLgar5 family transposase) which carried the *mecA* gene.

### Resistome and Virulome

A number of antibiotic resistance-associated genes were identified by using the Comprehensive Antibiotic Resistance Database (CARD). Table 3 shows the predicted antibiotic-resistance genes, the mechanism of resistance as well as the drug class for each isolate. All of the isolates except for C33 had at least one hit of the antibiotic resistant genes. All the resistome and virulome genes were located on the chromosomes except for *FosB* in isolate T28b which was located on the plasmid as well as *mecA* in isolate T20 which was located on an insertion sequence.

The Virulence Factors of Pathogenic Bacteria Database (VFDB) was used to determine the potential virulome of the isolates. All the isolates had at least one virulence factor (Table 4a and 4b). Interestingly, the analysis showed that some of the virulence factors were acquired from non-*Staphylococcus spp* (Table 4b) suggesting the possibility of these factors being acquired through horizontal gene transfer. The factors covered a broad range of categories which include adherences, enzymes, immune invasion, toxin, surface protein anchoring, anti-phagocytosis, and phagosome arrest among other categories.

## Discussion

In the current study, whole-genome sequencing of eight coagulase-negative staphylococci (CoNS) isolates was performed to characterise the antibiotic resistance and virulence factors associated with the isolates and the potential dangers they may pose. The isolates were from two distinct geographical locations (Supplementary Table 1) in which cadmium pollution and abuse of antibiotics were reported, thus we hypothesised that these anthropogenic activities are likely to influence the genomic features of the isolates. The pan-genomic approach was applied to highlight the variation of the isolates. Five of the eight isolates (T27, T28, T28b, C33, and D13b) were identified as *S. cohnni* (Fig. 1 and Table 2). Of these isolates, C33, D13b, and T28b were isolates from Nigeria, the remaining (T27, T28) were from South Africa. Isolates from South Africa (T20, T27, and T28) carried the tetracycline-resistant ribosomal protection protein (tetM) which was not detected from any of the samples from Nigeria. Phylogeographic structure, regional or continental endemism have been demonstrated in a number of bacterial species 32, 33. This correlates with the accessory genomes which are regarded as essential for survival under various selective pressures. In the current study, the* tetM* gene was part of the accessory genome, large fraction of the accessory genome are mobile genetic elements 34. These accessory genomes place the host cell in an advantageous position to be viable under specific conditions 35, 36. The elevated levels of tetracycline and other antibiotics in the wastewater treatment plants in the North West province, South Africa 12, could have exerted a selective pressure, which resulted in the acquisition of the *tetM* accessory genome probably through horizontal gene transfer. This presumption is also supported by the presence of fewer barriers to horizontal gene transfer among the genomes.

Environmentally driven heterogeneity among bacteria represents the physiological response to stress 37, and survival strategies developed over evolutionary time 38. All *S. cohnii* isolates from Nigeria possessed at least one gene for cadmium tolerance or resistance in their accessory genomes or singletons. Cadmium is one of the major driving forces for bacterial selection and evolution in the area where the isolates were collected (Nigeria). A number of studies have reported extreme soil and surface water contamination with heavy metals (including cadmium) in and around Ile-Ife, Nigeria 13, 14, 15, 16. The genomic heterogeneity among the samples from South Africa and Nigeria, clearly highlights how anthropogenic activity can shape the genomes of microbes in our surroundings.

**Figure 3:**
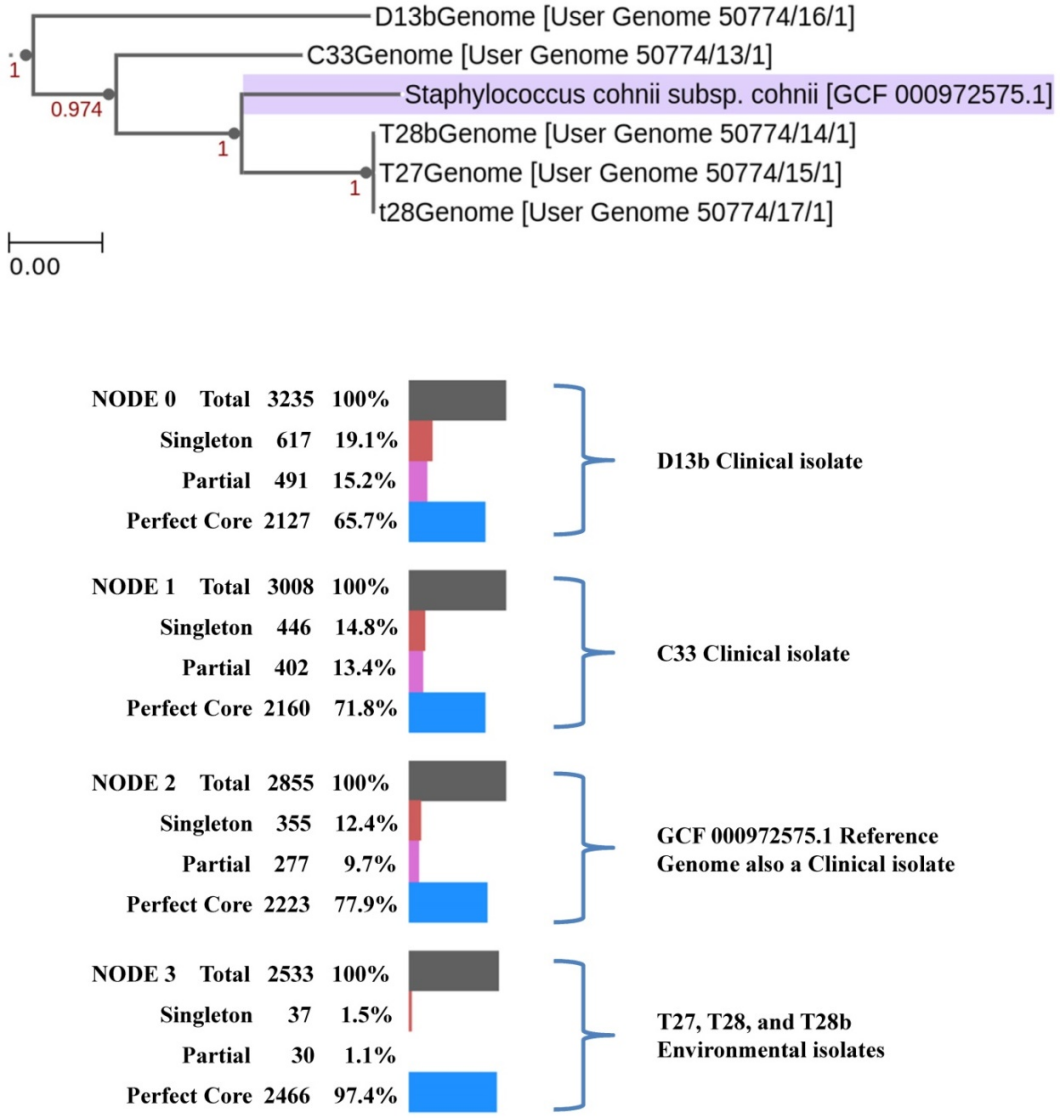
** Phylogenetic pan-genome accumulation of the *S. cohnni* isolates used in this study and the reference genome *S. cohnni* subsp cohnii (GCF_000972575.1).** Changes in the pan-genome are highlighted per each node.

Isolates used in this study possess an open pan-genome, increasing the number of species in the analysis resulted in an increase in the number of distinct genes (Fig. 3). Most pathogenic bacteria possess an open pan-genome, with the accessory genomes mainly consisting of virulent determinants and antibiotic-resistant genes on mobile genetic elements (MGEs) which would have been acquired through horizontal gene transfer. MGEs normally confers a number of adaptive advantages under a particular environment and support the virulence of organisms 39. In CoNS, MGEs have been shown to bear *S. aureus* pore-forming toxins and super-antigen enterotoxin coding sequences 40, which have been shown to modulate the virulence of CoNS bacteria 41. Isolates C33 and D13b had a significantly higher proportion of singletons compared to isolates T27, T28, and T28b. This was expected since the isolates were of clinical origin, and the singletons were presumed to be factors important for infection and hospital adaptation. True to this, the singletons consisted of virulence factors such as integrase, super-antigen encoding pathogenicity islands (SaPI), antimicrobial resistance (Undecaprenyl-diphosphatase (EC 3.6.1.27), PBP2a, MecA, MecR1, Blal, aadA), adaptations to stress (cadmium resistance protein, EfeB, abortive phage resistant protein, putative cytoplasmic protein).

Only one (T20) out of the eight isolates used in this study possessed the CRISPR/Cas system which is a strong barrier to foreign DNA uptake, in particular plasmid DNA 42, 43, 44. Consistent with this, T20 had no detected phages or plasmids (Table 1) however, T20 consisted of SaPIs, leading to the hypothesis that this pathogenic island might have been acquired before acquisition of the CRISPR/Cas region. CoNS have been shown to harbour fewer CRISPR/Cas systems compared to other bacteria 45. This has been linked to a potential role as a reservoir for antibiotic-resistant genes for *S. aureus* 46 since they can easily acquire these genes from the environment and transfer them to *S. aureus* through horizontal gene transfer. Intact prophages were only detected in isolates (L4 and C33). Prophages are known to increase the virulence potential of pathogenic strains 47, 48, and there is a positive correlation between the phage-related DNA content of a given Enterobacteria and its pathogenicity 49. In this study, phage-related DNA constituted a significant portion of the singletons among isolates, this was expected particularly in isolates of clinical origin.

CoNS are important reservoirs for antibiotic-resistant genes, including ß-lactamase genes. These are mainly carried on mobile genetic elements and can easily be transferred to highly virulent species such as *S. aureus* 50. The continuous spread of Methicillin-Resistant Staphylococcus aureus (MRSA) and Methicillin-Resistant Coagulase Negative Staphylococci (MR-CoNS) strains in clinical and non-clinical environments remain a serious public health concern. This is mainly due to the difficulties associated with the treatment of infections from such organisms as they become resistant to methicillin which is probably considered the prototype of anti-staphylococci penicillin 51. Methicillin resistance is characterised by an altered penicillin-binding protein (PBP2a) which has a reduced affinity for methicillin rendering the drug ineffective 4, 52, 53. PBP2a is encoded by mecA which is normally acquired through horizontal gene transfer of a mobile genetic element staphylococci cassette chromosome mec (SCCmec) 54. In the present study, mecA was detected only in isolate T20 designated as *S. lentus* (Table 3). The isolate also had multiple other antibiotic resistance genes which included tetracycline-resistant ribosomal protection protein (tetM), macrolide phosphotransferase (mphC) Erm 23S ribosomal RNA methyltransferase(s) (Erm43/A/B). This augments the ever-increasing evidence of wastewater treatment plants being a source of multiple antibiotic resistance genes. A diverse group of antibiotics targets the ribosome as the major site of action in the bacterial cell 55, thus altering and protecting these target sites is vital for bacterial survival in the face of antibiotics. Methylation of rRNA provides acquired antibiotic resistance 56, thus the Erm group provides a crucial role in acquired resistance. Tetracycline, a broad-spectrum antibiotic agent binds to elongating ribosome and inhibit delivery of the ternary complex to the A-site 57 thus inhibiting bacterial translation and slowly killing it. Continuous use and abuse of tetracycline resulted in bacteria acquiring resistance determinants to this group of antibiotics 58. The resistance determinants mainly include ribosome protection proteins with the most prevalent being tetM and tetO 59, 60. The tetM was also detected in *S. cohnii* isolates (T27 and T28; Table 3). Also of interest was the detection of genes associated with ß-lactamase activities. The isolates identified as *S*. *haemolyticus* carried the PC1 ß-lactamase (blaZ) gene (Table 3). Table 3 gives an overview of the detected genes providing resistance to various antibiotics. This table also provides information on drug class and resistance mechanism.

Eighty percent of the *S. cohnii* isolates contained the *FusF* gene. These were from both Nigeria and South Africa. Resistance to fusidic acid is selected for by topical antibiotic use. An increase in the emergence of fusidic acid resistance among clinical isolates which were attributed to the widespread and inappropriate use of fusidic acid cream for chronic skin conditions has been recently reported 61. Normally, resistance is conferred by genes encoding FusB-family proteins through the protection of the drug target site 62. However, Chen et al., 63 identified a novel fusidic acid resistance determinant, FusF, in fusidic acid resistant *S. cohnii*. The gene is thought to be a natural part of the *S. cohnii* genome. From the study, only *S. cohnii* carried this gene out of a number of CoNS species, and this was similar to the present study.

## Conclusion

In conclusion, anthropogenic activities seemed to impact the accessory genome, contributing to the variation of isolates within the same species. CoNS isolates used in this study possessed an open pan-genome with few barriers for horizontal gene transfers. Thus the isolates could easily acquire foreign DNA, which helps to adapt to the selective pressure, this explains the observed environmentally driven heterogeneity. Pan-genome analysis proved to be a powerful tool for comparative genomics as it helped to highlight the environmentally driven heterogeneity of the isolates. Moreover, the clinical isolates could be clearly distinguished from the environmental isolates based on the composition of the singletons. More interestingly, was the presence of virulence determinants and multiple antibiotic resistance genes from environmental isolates, demonstrating the importance of wastewater treatments as a source of antibiotic resistant genes. This study adds to the baseline for antibiotic resistance surveillance and comparative genomics of potentially pathogenic CoNS.

## Supplementary Material

Supplementary figures and tables.

## Figures and Tables

**Table 1:** General features of the genomes including the assembly metrics.

	L4	D13b	T6	T20	C33	T28	T28b	T27
Genome Size (bp)	2,135,828	2,685,820	2,299,081	2,648,007	2,576,421	2,643,319	2,690,294	2,648,416
G+C Content %	32.78	32.55	32.64	31.82	32.39	32.36	32.37	32.33
Number of Contigs	60	22	49	36	37	12	12	14
Largest Contig	247,342	984,404	278,121	200,372	434,148	1,296,694	1,298,284	1,296,793
N50	41,586	252,300	76,220	121,032	131,536	563,483	566,475	563,483
Genes	2,162	2,658	2,284	2,619	2,497	2,544	2,603	2,551
Protein coding gene	2,087	2,626	2,244	2,601	2,458	2,499	2,540	2,504
Plasmids	0	1	1	0	0	2	3	2

**Table 2:** Average Nucleotide Identity (ANI) of the isolates.

Query Genome	Reference Genome	ANI estimate	Total orthologous sequence fragments matched	Total sequence fragments of query
T28	GCF_000972575.1_assembly	99.1921	816	874
	(S. cohnii subsp. cohnii)			
T20	GCF_000286395.1_assembly	99.1546	743	863
	(S. lentus F1142)			
T27	GCF_000972575.1_assembly	99.1311	812	874
	(S. cohnii subsp. cohnii)			
T28b	GCF_000972575.1_assembly	99.0809	821	890
	(S. cohnii subsp. cohnii)			
C33	GCF_000972575.1_assembly	98.9739	795	838
	(S. cohnii subsp. cohnii)			
T6	GCF_000009865.1_assembly	98.8268	718	744
	(S. haemolyticus JCSC1435)			
L4	GCF_000009865.1_assembly	98.7875	656	685
	(S. haemolyticus JCSC1435)			
D13b	GCF_000972575.1_assembly	98.7468	805	884

**Table 3:** Antibiotic resistant genes identified from the isolates.

Isolate	RGI Criteria	ARO term	AMR Gene Family	Drug Class	Resistance Mechanism	Matching Region	Ref Seq Length
T20	perfect	*mec*l	methicilin resistant PBP2	penam	antibiotic target replacement	100	100
	Strict	*tetM*	tetracylin-resistant ribosomal protection protein	tetracyclin antibiotic	antibiotic target protection	97.65	100
	Strict	*mecA*	methicilin resistant PBP2	penam	antibiotic target replacement	99.85	100
	Strict	*mphC*	macrolide phosphotransferase (MPH)	macrolide antibiotic	antibiotic inactivation	90.64	100
	Strict	*Erm(43)*	Erm 23S ribosomal RNA methyltransferase	macrolide antibiotic, lincosamide antibiotic, streptogramin antibiotic	antibiotic target alteration	98.77	100
	Srict	*ErmA*	Erm 23S ribosomal RNA methyltransferase	macrolide antibiotic, lincosamide antibiotic, streptogramin antibiotic	antibiotic target alteration	99.59	100
	Strict	*ErmB*	Erm 23S ribosomal RNA methyltransferase	macrolide antibioyic, lincosamide antibiotic, streptogramin antibiotic	antibiotic target alteration	98.78	98.79
L4	Perfect	*dfrG*	trimethoprim resistant dihydrofolate reductase dfr	diaminopyrimidine antibiotic	antibiotic target replacement	100	100
	Strict	PC1 beta- lactamase (*blaZ*)	blaZ beta lactamase	penam	antibiotic inactivation	95.37	100
d13b	Strict	*FusF*	fusidic acid inactivation enzyme	fusidic acid	antibiotic inactivation	99.07	100
T28b	Perfect	*FosB1*	fosfofomycin thiol transferase	fosfomycin	antibiotic inactivation	100	100
	Strict	*FusF*	fusidic acid inactivation enzyme	fusidic acid	antibiotic inactivation	99.53	100
	Strict	*FosD*	fosfofomycin thiol transferase	fosfomycin	antibiotic inactivation	96.4	100
T27	Srict	*FusF*	fusidic acid inactivation enzyme	fusidic acid	antibiotic inactivation	99.53	100
	Strict	*tetM*	tetracylin-resistant ribosomal protection protein	tetracyclin antibiotic	antibiotic target protection	97.81	100
T6	Strict	PC1 beta-lactamase (*blaZ*)	blaZ beta lactamase	penam	antibiotic inactivation	96.09	100
T28	Strict	*FusF*	fusidic acid inactivation enzyme	fusidic acid	antibiotic inactivation	99.53	100
	Strict	*FosD*	fosfofomycin thiol transferase	fosfomycin	antibiotic inactivation	96.4	100
	Strict	*tetM*	tetracylin-resistant ribosomal protection protein	tetracyclin antibiotic	antibiotic target protection	97.81	100
C33	n/a	n/a	n/a	n/a	n/a	n/a	n/a

**Table 4a:** Distribution of virulence factors which are largely confined to the Staphylococcus genus.

		L4	D13b	T28	T28b	C33	T20	T6	T27	S. aureus (subsp aureus)
**Adherence**										
Autolysin	(*atl*)	+	+	+	+	+	-	+	+	SACOL1062
Clumping factor B	(*clfB*)	+	-	-	-	-	+	-	-	SACOL2652
Elastin binding protein	(*ebp*)	+	+	+	+	-	-	+	+	SACOL1522
**Enzymes**										
Lipase	(*lip*)	+	+	+	+	-	-	-	+	SACOL2694
Serine V8 protease	(*sspA*)	-	+	+	+	+	+	+	+	SACOL1057
Thermonuclease	(*nuc*)	+	+	+	+	+	-	+	+	
**Immune invasion**										SACOL0860
Capsule		+(2)	+(5)	+(6)	+(6)	-	+(3)	+(2)	+(6)	SACOLO136 -141

**Table 4b:** Distribution of virulence factors likely to have been acquired from non-Staphylococcus genomes.

		L4	D13b	T28	T28b	C33	T20	T6	T27	Organism
**Imune invasion**										
Capsule		-	-	+	+	-	-		+	Acinetobacter
Polysacharide capsule		-	-	-	-	-	+		-	Bacilus
**Toxin**										
Cytolisin	(*cyIR2*)	-	-	+	+	+	-		+ (4)	Enterococcus
**Nutrional factor**										
Allantoin utilisation		-	+ (4)	+ (4)	+ (4)	+ (4)	-		+	Klebsiela
**Serum resistance and immune invasion**										
LPS	(*wbtE*)	-	+	-	-	-	-		-	(Francisella)
LPS	(*wbtP*)	-	-	-	-	-	+		-	(Francisella)
**Antiphagocytocysis**										
Capsule	(*uge*)	-	-	+	+	-	-		+	Klebsiela
**Iron uptake**										
Periplasmic binding protein-dependent ABC transport systems	(*vctC*)	-	-	-	-	-	+		-	Vibrio
**Phagosome arresting**										
Nucleoside diphosphate kinase	(*ndk*)	-	-	-	-	-	+		-	Mycobacterium
**Regulation**										
LisR/LisK	(*lisR*)	-	-	-	-	-	+		-	Listeria
**Surface protein anchoring**										
Lipoprotein diacylglyceryl transferase	(*lgt*)	-	-	-	-	-	+		-	Listeria
