# Supplementary material for: Analysis of Genome Sequences of Coagulase-Negative Staphylococci Isolates from South Africa and Nigeria Highlighted Environmentally Driven Heterogeneity
Source: J Genomics. 2021 May 13;9:26–37. doi: 10.7150/jgen.53019 (PMC8133835; doi:10.7150/jgen.53019)
Supplement: Supplementary file 1 — Supplementary figures and tables. [file jgenv09p0026s1.pdf]

Supplementary Table 1. Description of the isolates origins

Isolate/Sample ID	Source	Identity	Country of Origin	Settings
T20	WWTP	*S. lentus*	South Africa	Environmental
T27	WWTP	*S. cohnii*	South Africa	Environmental
T28	WWTP	*S. cohnii*	South Africa	Environmental
T6	WWTP	*S. haemolyticus*	South Africa	Environmental
T28b	WWTP	*S. cohnii*	Nigeria	Environmental
C33	Wound Swab	*S. cohnii*	Nigeria	Clinical
L4	Wound Swab	*S. haemolyticus*	Nigeria	Clinical
D13b	Urine	*S. cohnii*	Nigeria	Clinical

WWTP: Waste Water Treatment Plant

Supplementary Table 2. CheckM quality assessment of the assembled genomes

Isolate	Completeness (%)	Contamination (%)
T28	99.45	0
T28b	99.45	0
T27	99.45	0
T6	98.34	0.55
D13b	96.45	0.55
L4	91.91	0.55
C33	99.45	1.1
T20	98.34	1.1

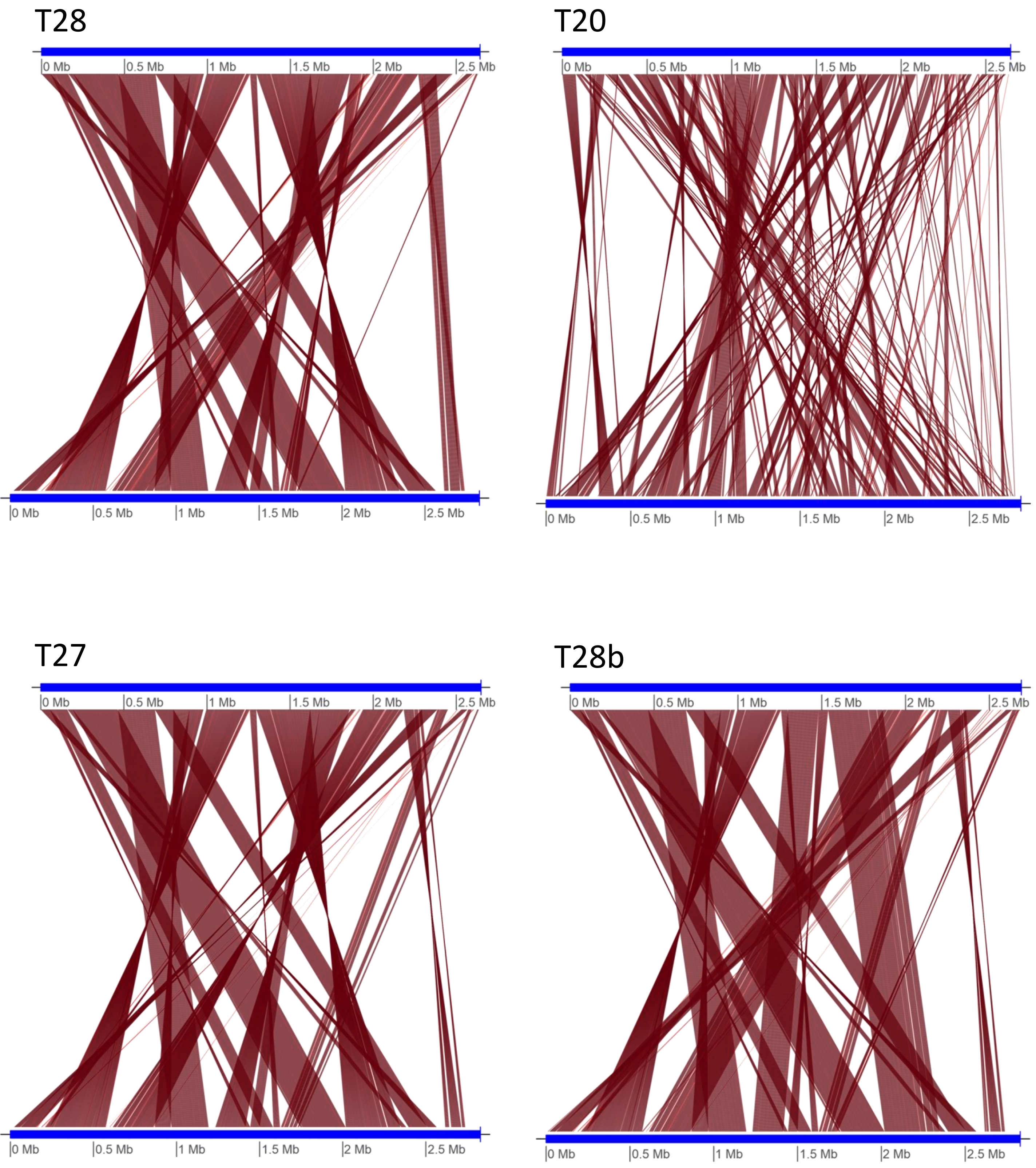

Supplementary Fig.1

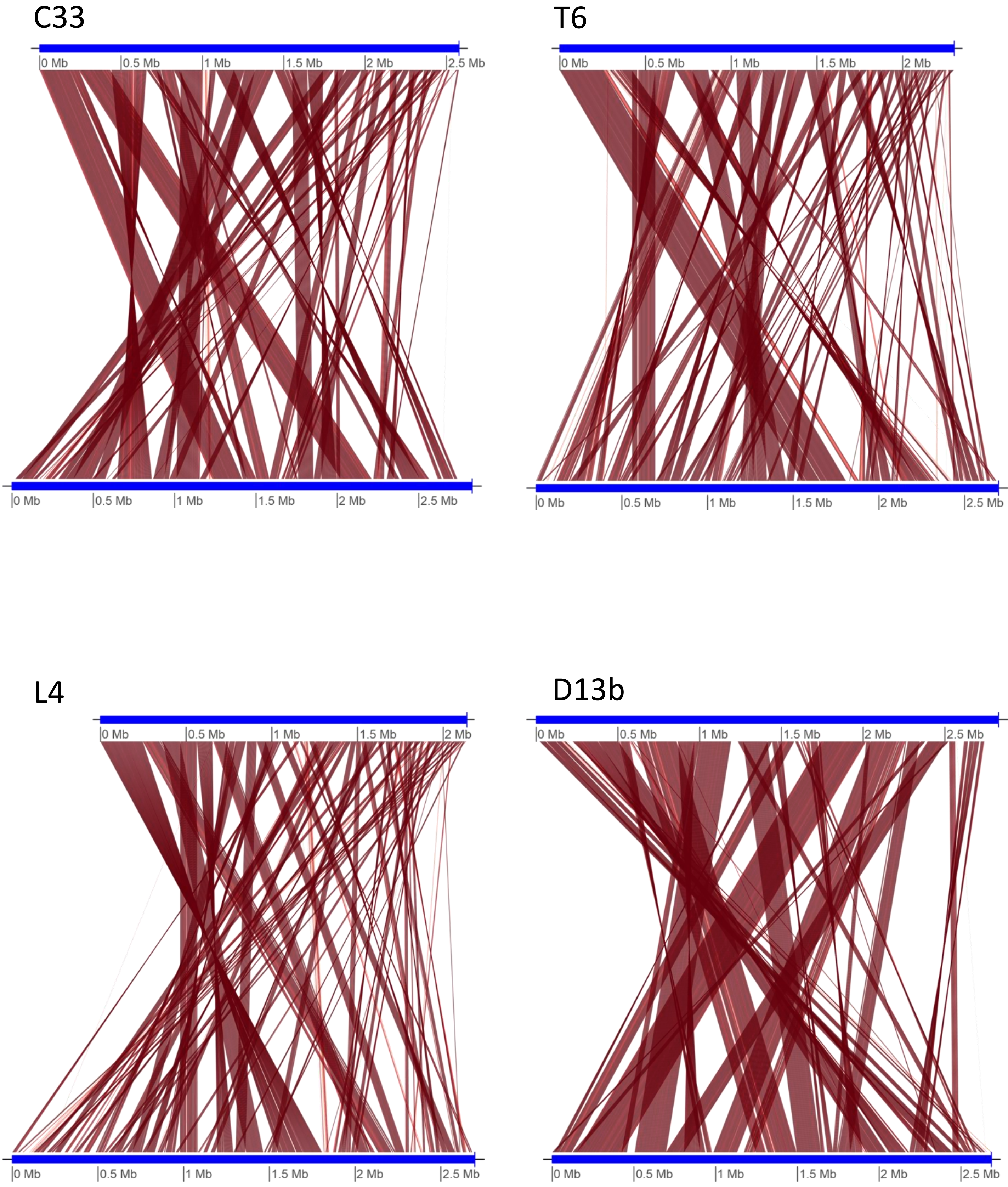

Supplementary Fig.1
